# Toxicity and Residual Effect of Toxic Baits on Adults of *Spodoptera frugiperda* (Lepidoptera: Noctuidae): Implications for Pest Management

**DOI:** 10.3390/insects17010108

**Published:** 2026-01-18

**Authors:** José Gomes da Silva Filho, Otavio Ribeiro Duarte, Paloma Stüpp, Júlia Peralta Ferreira, Lígia Caroline Bortoli, Juarez da Silva Alves, Larissa Pasqualotto, Michele Trombin de Souza, Mireli Trombin de Souza, Vanessa Nogueira Soares, Juliano Pazini, Leandro do Padro Ribeiro, Ruben Machota Junior, Daniel Bernardi

**Affiliations:** 1 Department of Plant Protection, Federal University of Pelotas (UFPel), Capão do Leão 96010-610, Rio Grande do Sul, Braziljulianopazzini@hotmail.com (J.P.); 2 ISCA Tecnologias, Ijuí 98700-000, Rio Grande do Sul, Brazil; 3 Research Center for Family Agriculture, Agricultural Research and Rural Extension Company of Santa Catarina (CEPAF/EPAGRI), Chapecó 89801-970, Santa Catarina, Brazil

**Keywords:** food attractant, attract-and-kill system, lepidoptera, IPM, behavioral control

## Abstract

*Spodoptera frugiperda* is a major agricultural pest both in Brazil and worldwide, with widespread resistance to synthetic insecticides. This study evaluated the toxicity and residual activity of toxic bait formulations combining 17 insecticides with the plant-based kairomone attractant Noctovi^®^ 43SB against *S. frugiperda* adults. Bioassays were conducted with 48 h-old, food-deprived adults. Toxicity was assessed by incorporating insecticides at 2% concentration into Noctovi^®^ 43SB. Residual activity was measured by applying toxic baits on cotton leaves and testing at 3, 7, 15, 21, and 30 days after application (DAA). Insecticides based on methomyl, spinetoram, spinosad, indoxacarb, malathion, and zeta-cypermethrin induced over 95% mortality. Methomyl showed the greatest toxicity (LC_50_ = 322 mg a.i. L^−1^; LC_90_ = 1160 mg a.i. L^−1^). Eleven insecticides maintained residual activity (≥70%) after 30 DAA, except malathion. Overall, toxic bait formulations combining Noctovi^®^ 43SB with methomyl, spinosad, spinetoram, or indoxacarb proved highly effective, offering both rapid and prolonged control of *S. frugiperda* adults. These findings highlight attract-and-kill as a promising tool for sustainable management of this pest.

## Simple Summary

*Spodoptera frugiperda* is considered a major pest in commodity production systems (soybean, maize, and cotton), causing severe economic losses. The main management tools rely on the use of synthetic insecticides and *Bt* crops. However, their frequent use has led to resistance issues and reduced control efficacy. To overcome these challenges, new management strategies based on behavioral control (attract-and-kill) have been investigated. This study evaluated synthetic insecticides in toxic bait formulations and their residual activity against adult *S. frugiperda*. The results showed that the food attractant Noctovi^®^ 43SB combined with the insecticides methomyl, spinetoram, spinosad, and indoxacarb achieved mortality rates above 90%. In addition, the tested insecticides exhibited high residual activity, maintaining mortality rates above 70%, even 30 days after application. The use of attract-and-kill management strategies proved to be promising, providing high pest mortality and extended residual activity. Furthermore, toxic bait formulations allow for reduced insecticide use in cultivated areas, while also contributing to the maintenance of susceptible individuals and local biodiversity.

## 1. Introduction

*Spodoptera frugiperda* (J.E. Smith) (Lepidoptera: Noctuidae) is an important insect pest due to both its voracity and polyphagy; utilizing at least 353 plant species as host [1]. *Spodoptera frugiperda* has a preference for plants from the Poaceae family and is considered a key pest of maize (*Zea mays* L.; Poaceae) [2]. However; in the absence of plants from the Poaceae family; *S. frugiperda* will feed on other agriculturally relevant plant species; such as cotton (*Gossypium hirsutum* L.; Malvaceae) and soybean (*Glycine max* (L.) Merril; Fabaceae); especially when these crops are planted in succession to maize [3,4]. Factors associated with its biological potential; including the production of multiple generations per year and high reproductive and dispersal capacity, contribute to its establishment and damage to crops [5]. Native to the Americas, *S. frugiperda* is distributed in several countries, such as Brazil, Argentina, Mexico and the United States [2,6,7]. Lately, the pest has been spread to African [8,9] and Asian continents [10,11] and Oceania [12], and more recently, to Europe [13].

To reduce the damage caused by this pest species, various control methods are used, especially synthetic insecticides spraying and transgenic technologies that express insecticidal proteins derived from *Bacillus thuringiensis* (*Bt* events) [9,14]. However, the misuse of these technologies has led to an increase in cases of resistant populations, that compromise the effectiveness of these management tools [15,16]. Several cases of *S. frugiperda* developing resistance to chemical groups of insecticides have already been reported, including pyrethroids, organophosphates, benzoylureas, spinosyns and anthranilic diamides [15,17,18,19,20,21]. The pest has also evolved resistance to different *Bt* events, including those that express the proteins Cry1F [5,22], Cry1Ab [23], Cry1A.105 + Cry2Ab2 [24] and Vip3Aa [25,26].

In light of this context, the development of new management tools has gained importance in the framework of Integrated Pest Management (IPM) [27]. Semiochemicals have been used for monitoring programs, mass capture and sexual disruption of male individuals in cultivation areas [28,29,30]. Among the approaches that use semiochemicals as a basis for attraction and control, the “attract-and-kill” system is a direct-action control tool that aims to remove male and female individuals from the growing area [31,32,33,34,35]. It helps to reduce or eliminate the spraying of insecticides on the entire crop area, reducing the potential harmful effects on beneficial agents [36,37].

Various studies are being carried out to formulate “attract-and-kill” systems and, currently, the food attractant Magnet^®^ has been used in Australia in a mixture with the insecticide thiodicarb to manage *Helicoverpa armigera* (Hübner) (Lepidoptera: Noctuidae) in cotton plants [37,38]. Likewise, in China, farmers have been using a formulation based on kairomones known as Bioattract Heli^®^ infused with the insecticide methomyl, which has shown a reduction in population density and a reduction in damage by *H. armigera* immatures in cultivated areas [39]. In Brazil, the use of toxic baits has been studied for the management of *H. armigera*, *Chloridea virescens* (Fabricius) and *S. frugiperda* using the attractants Chamariz^®^ (AgBitech) and Noctovi^®^ 43SB (Iscas Tecnologias) and in a mixture with a killing agent (insecticide) that promotes rapid mortality (knock-down) of adults insects [40]. The Noctovi^®^ 43SB is a liquid food-based attractant designed for use in “attract-and-kill” systems. The attractant was developed as a blend of plant-derived kairomones [(Z)-11-Hexadecenal (Z11-16: Ald), (Z)-9-Hexadecenal (Z9-16; Ald)] combined with other phagostimulants, such as sugars and proteins. This formulation aims to attract both sexes of Lepidoptera species within the family Noctuidae [41,42].

Another important factor in the formulation of toxic baits is the killing agent (insecticides). The killing agents used in toxic bait formulations should possess characteristics that promote rapid incapacitation of adult insects (mortality), thereby reducing the likelihood of adults—particularly females—flying away from the ingestion area to mate or oviposit [43,44]. Insecticides with neurotoxic modes of action, such as carbamates (methomyl), organophosphates (chlorpyrifos, malathion, profenofos + cypermethrin), pyrethroids (zeta-cypermethrin, lambda-cyhalothrin), oxadiazines (indoxacarb), pyrazoles (fipronil), and pyrazole analogs (chlorfenapyr), among others, have been identified as promising candidates for toxic bait formulations due to their rapid action [45]. Moreover, other insecticide chemical groups, such as spinosyns (spinosad, spinetoram), avermectins (emamectin benzoate), and anthranilic diamides (chlorantraniliprole), although exhibiting slower lethal effects on insects, contribute to reducing key behavioral activities such as long-distance flight, feeding, mating, and oviposition. These effects can significantly influence pest suppression and population dynamics [46,47].

Although effective, toxic baits can lose their toxicity to pests due to various factors, including: (1) rainfall events, which promote the physical removal of the bait by the impact of the raindrop [48]; (2) rapid loss of attractiveness of the volatile compounds [49] and (3) degradation of the active ingredient due to the action of solar radiation [50]. Thus, the present study aimed to evaluate the efficacy of synthetic insecticides commonly used for the management of immature stages of *Spodoptera frugiperda* when incorporated into toxic bait formulations, as well as their residual activity in conditions greenhouse. Given this scenario; we assessed the toxicity of insecticides with different modes of action in formulations with the food attractant Noctovi^®^ 43SB as well as evaluated the residual effect on *S. frugiperda* adults when applied to cotton leaves (greenhouse trial). These results are part of ongoing efforts to provide research-based insight into the performance of toxic baits. This knowledge is important to growers and other stakeholders in their efforts to optimize insecticide applications, avoid widespread spraying, and reduce the risk of insect resistance when used in conjunction with other management tactics.

## 2. Materials and Methods

### 2.1. Insects

The insects used in the bioassays came from a population of *S. frugiperda* collected on non-*Bt* corn during the 2021/2022 harvest in Campo Verde, MT, Brazil (15°18′34.64″ S; 54°53′47.681″ W). The population of *S. frugiperda* was maintained in laboratory conditions with temperatures of 25 ± 2 °C, relative humidity of 70 ± 10% and 12 h photophase for 18 generations (approximately 2 years) on an artificial media based on white beans, wheat germ and yeast [51]. The adult insects were kept in PVC cages lined with recyclable office paper as an oviposition substrate and fed with a 10% (*v v*^−1^) honey solution.

### 2.2. Products Test

The insecticides used to formulate the toxic bait are described in Table 1. Conversely, the commercial food attractant Noctovi^®^ 43SB were provided by ISCA Technologies (Riverside, CA, USA).

### 2.3. Toxicity of Insecticides in Toxic Bait Formulations

The bioassays were carried out using 500 mL polyethylene terephthalate (PET) plastic cages. Six adults (three couples) of *S. frugiperda* were placed in each cage at 48 h of age and deprived of food for 24 h. To formulate the toxic bait, different insecticides (Table 1) were used at a concentration of 2% of active ingredient of the commercial product (p.c.) in a mixture with the undiluted food attractant Noctovi^®^ 43 SB [42]. This concentration was defined based on manufacturer recommendation [42].

After obtaining the solution, a drop of 100 µL was placed on a 4.0 cm^2^ plastic plate and offered to *S. frugiperda* adults during the scotophase period; i.e; at 18:00 p.m. and removed at 06:00 a.m. After 12 h of offering, the baits were removed and the *S. frugiperda* adults were fed with a 10% (*v v*^−1^) honey solution in hydrophilic cotton offered in 20 mm diameter plastic containers. Insects fed only on the food attractant Noctovi^®^ 43SB were used as a negative control.

The experimental design adopted was completely randomized, with five replicates (three *S. frugiperda* couples per replicate) per treatment. The mortality of *S. frugiperda* adults was assessed at each 24 h after exposure for 120 h. Individuals which did not show movement equivalent to the length of their body were considered dead.

### 2.4. Curves of Dose–Response of Toxic Baits to S. frugiperda Adults

The most promising insecticides found in the previous bioassay were studied again to estimate the LC_50_ and LC_90_ values (lethal concentrations needed to kill 50% and 90% of *S. frugiperda* adults, respectively). For this purpose, five concentrations were defined: 0.10, 0.25, 0.5, 1.0, 1.5, 1.75 and 2.0%. The exposure time, evaluation procedures and criteria were identical to those used in the toxicity tests. The experiment was completely randomized with five replicates per concentration, being each replicate constituted by six *S. frugiperda* adults. The mortality of *S. frugiperda* adults was assessed at 24 h intervals for 120 h. Individuals which did not show movement equivalent to the length of their body were considered dead. All the bioassays were conducted in air-conditioned rooms with temperatures of 25 ± 2 °C, relative humidity of 70 ± 10% and a 12 h photoperiod.

### 2.5. Residual Effect of Toxic Baits on S. frugiperda Adults (Greenhouse Trial)

For this purpose, cotton seedlings cv. TMG44B2RF were used, grown in plastic pots with a capacity of 5 L (one plant per pot) and kept inside a greenhouse (temperature 25 ± 1 °C; RH 47 ± 2% and photophase 12 h). At phenological stage B1 (the beginning of the first visible bud), 100 µL drops of the bait formulations were applied to the adaxial side of the leaves using a 1000 µL automatic single-channel micropipette (HTL^®^ SA; Warsaw, Mazovia, Poland).

The toxic bait formulations (treatments) were made using 12 insecticides at a concentration of 2.0% of the commercial product (c.p). in a mixture with the food attractant Noctovi^®^ 43SB. After applying the treatments, cotton leaves containing a drop of the solution were removed from the plant and offered to *S. frugiperda* adults after 3, 7, 15, 21 and 30 Days After Application (DAA). For this purpose, the leaves treated with the baits were detached from the plant and taken to the laboratory. In the laboratory, the leaves were cut into a disk (4 cm^2^) in the area of the application drop and placed in a plastic container with a diameter of 2.0 cm covered with a piece of moist absorbent cotton. Subsequently, the leaf disks were placed in a humid chamber for 1 h to hydrate the formulation (simulating night dew) and then fed to adult *S. frugiperda* insects at 48 h of age and deprived of food for 24 h.

For each evaluation date (DAA), the baits were supplied during the scotophase from 6 pm to 6 am. After 12 h, the baits were removed and the insects were fed with a 10% mead solution as described previoulsy. As a negative control, the insects were exposed only to mead solution. The experiment was conduct under a completely randomized design with 12 treatments (toxic bait formulations), each treatment consisting of 5 replicates (six *S. frugiperda* couples per replicate). Mortality was assessed daily for a period of 5 days. Individuals that did not show movement equivalent to their body length were considered dead.

### 2.6. Data Analysis

For all bioassays (toxicity and residual effect), *S. frugiperda* adult mortality data were submitted to the Shapiro–Wilk normality test (SPSS Inc., Chicago, IL, EUA). A binomial model with a complementary log-log link function (gompit model) was used to estimate the lethal concentrations (LC_50_ and LC_90_) using the Probit Procedure in the software SAS, version 9.2 [52]. A probability test (F-test) was conducted to test the hypothesis that the LC values were equal. If the hypothesis was rejected, pairwise comparisons were performed, and significance was assumed when there was no overlap of the confidence intervals. For the evaluation of the toxicity of the toxic baits and the residual effect, the data on the survival rates of the *S. frugiperda* adults that did not present a normal distribution were transformed with a Box–Cox transformation prior to the analyses. Subsequently, a two-way analysis of variance was performed on all the data using PROC GLM. The differences between the treatments were determined by the least-squares means (PDIFF option in PROC GLM) followed by Tukey’s adjustment based on a 5% significance [52].

## 3. Results

**Figure 1:**
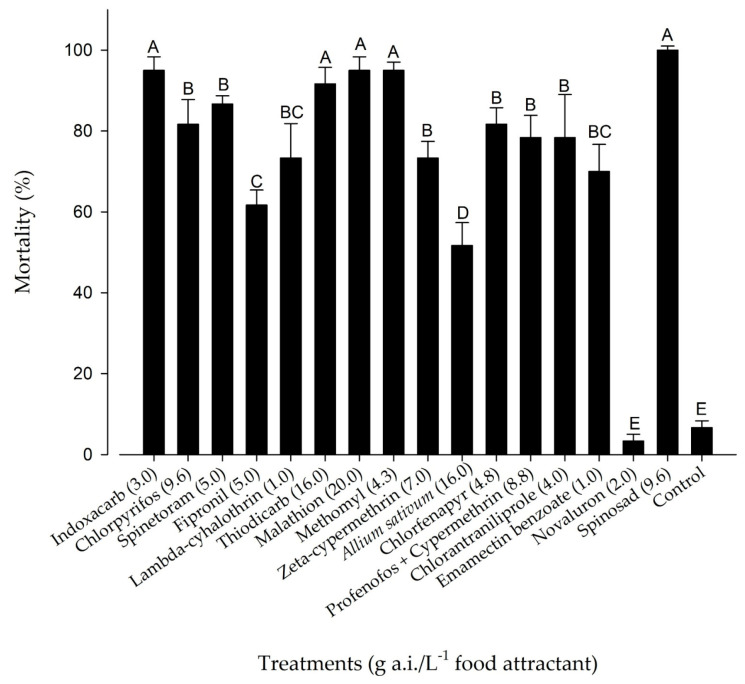
Mortality (%) of *S. frugiperda* adults exposed to toxic bait formulations with the food attractant Noctovi^®^ 43SB in the laboratory conditions (temperatures of 25 ± 2 °C, relative humidity of 70 ± 10%*)*. Means (±SE) followed by same letters in the column do not differ statically by Tukey’s test; *p* < 0.05. Mortality corrected by the Henderson and Tilton [53] formula.

The insecticides used in the toxic bait formulations with the Noctovi^®^ 43SB attractant showed significant differences (F = 22.98; df = 16.84; *p* < 0.001). However, insecticides containing methomyl, spinetoram, spinosad, indoxacarb, malathion, and zeta-cypermethrin exhibited a mortality rate greater than 95% in *S. frugiperda* adults (Figure 1). On the other hand, the insecticides novaluron and fipronil showed mortality rate lower than 50% (Figure 1).

Based on the dose–response values, the toxic baits formulated with the insecticide based on methomyl (LC_50_ = 322.0 mg L^−1^ and LC_90_ = 1160.0 mg a.i. L^−1^), showed the highest toxicity on *S. frugiperda* adults, followed by the toxic baits formulated with the insecticide based on indoxacarb (LC_50_ = 810.0 mg L^−1^ and LC_90_ = 2610.0 mg a.i. L^−1^) and spinetoram (LC_50_ = 816.0 mg L^−1^ and LC_90_ = 3648.0 mg a.i. L^−1^) (Table 2). In contrast, based on the LC_50_ and LC_90_ values, toxic baits containing thiodicarb (LC_50_ = 7760.0 mg L^−1^ and LC_90_ = 16,800.0 mg a.i. L^−1^), spinosad (LC_50_ = 5280.0 mg L^−1^ and LC_90_ = 7296.0 mg a.i. L^−1^) and zeta-cypermethrin (LC_50_ = 3040.0 mg L^−1^ and LC_90_ = 7520.0 mg a.i. L^−1^) provided the lowest toxicities (Table 2).

In greenhouse experiments devoted to assess the residual effect, it was observed that the insecticides methomyl (F = 4.11; df = 4; 16; *p* < 0.001), spinosad (F = 6.77; df = 4; 16; *p* < 0.001), clofenapyr (F = 5.16; df = 4; 16; *p* < 0.001), spinetoram (F = 7.89; df = 4; 16; *p* < 0.001), and malathion (F = 9.10; df = 4; 16; *p* < 0.001) presented significant differences in the mortality rate of *S. frugiperda* adult insects on the studied aging dates (Table 3). When we study the effects of the residual activity of insecticides overtime, we find significant differences in the mortality of *S. frugiperda* adults between zeta-cypermethrin (80% of mortality) with the other insecticides that were tested after three days after application (DAA) (F = 12.45; df = 11; 55; *p* < 0.001) (Table 3), reaching 100% mortality with the use of the insecticides based on methomyl and spinosad. At 7 DAA (F = 17.11; df = 11; 55; *p* < 0.001) were founded that the zeta-cypermethrin (78.3%) and malathion (78.0%) showed significant differences in the mortality of the pest crop compared to the other insecticides studied.

During the 30 DAA period were observed significant difference in the mortality rate of *S. frugiperda* adults (F = 9.04; df = 11; 55; *p* < 0.001). Meanwhile, the insecticides chlofenapyr, emamectin benzoate, indoxacarb, chlorantraniliprole, thiodicarb, and lambda-cyhalothrin presented a mortality rate greater than 90% (Table 3). In turn, the insecticide methomyl (F = 4.11; df = 4; 16; *p* < 0.001) showed adult mortality of *S. frugiperda* > 80% at 30 DAA.

## 4. Discussion

Considered highly destructive to major agricultural commodities (soybean, maize, and cotton) worldwide, *S. frugiperda* has become increasingly difficult to manage due to its high fecundity and frequent reports of populations resistant to the main management technologies (insecticides and *Bt* crops) [5,15,54]. To minimize losses and delay the evolution of resistance, farmers have sought new, more sustainable integrated pest management tools, along with a reduced use of synthetic insecticides and selective products that spare non-target organisms. Management tools based on behavioral manipulation of pest insects have emerged as promising alternatives for controlling several species across different families, including Tephritidae (*Ceratitis capitata* (Wiedemann), *Anastrepha fraterculus* (Wiedemann)) [48,55], Plutellidae (*Plutella xylostella* (Linnaeus)) [56], Noctuidae (*S. frugiperda*, (J.E. Smith), *H. armigera* (Hübner)) [39,40], Curculionidae (*Anthonomus grandis* (Boheman)) [57], among others. Behavioral manipulation techniques have been employed with various objectives, such as mating disruption (sexual pheromones) [58], monitoring (light traps, color traps) [59,60], mass trapping (food-based attractants) [61], and attract-and-kill systems [34], all aimed at pest suppression and control. These strategies target the adult stage of the pest, thereby reducing reproductive output and suppressing crop damage [62].

Pest management based on attract-and-kill systems (toxic baits) has become an alternative tool to conventional control practices (insecticides). Moreover, the use of toxic baits has contributed to suppressing pest population peaks in *Bt* cropping systems under field conditions. The attract-and-kill system typically consists of attractive formulations (a wax-based matrix containing blends of plant volatile compounds or sexual pheromones) to lure the insect, combined with phagostimulant components (sucrose, fructose) and a mortality agent (synthetic insecticide), preferably with rapid knockdown action [54,62]. Insecticides acting on the nervous or muscular system (carbamates, organophosphates, pyrethroids, neonicotinoids, among others) have been the most recommended for use in toxic bait formulations due to their fast action in causing mortality after ingestion [63].

Lethal agents are considered essential components of toxic bait formulations, as variations in pest mortality may be associated with species, developmental stage, insecticide type, and concentration used [64,65]. According to these authors, it is important to highlight that most insecticides employed in toxic bait formulations are designed primarily based on the susceptibility of immature stages of the target pest. However, Bird and Drynan [61] and Leonova et al. [66] note that the efficacy of a given insecticide does not necessarily correlate across developmental stages, which is likely associated with inherited differences in susceptibility throughout the insect’s life cycle. In addition, Forrester et al. [67] discuss that adult insects exhibit weaker metabolic defenses, which consequently contributes to higher control efficiency when insecticides are ingested orally, due to their reduced capacity to express metabolic resistance. Although different levels of susceptibility occur among the developmental stages of the pest, this aspect was not addressed in the present study and warrants further investigation in future research.

In the present study, we found that candidate insecticides for use in toxic bait formulations exhibited variation in mortality rates; however, insecticides such as spinosad, methomyl, indoxacarb, thiodicarb, and emamectin benzoate produced ≥90% mortality. These results are consistent with those reported by [68], who observed 100% mortality for the insecticides Lannate^®^ (methomyl) and Success^®^ (spinosad) in toxic bait formulations offered to *H. armigera*. Similarly, Galm and Sparks [44] also reported 100% mortality of adult *Rhagoletis indifferens* (Rhagin) (Diptera: Tephritidae) when exposed to toxic bait formulated with sucrose and spinosad. According to Schultze et al. [69], toxic bait formulations composed of hydrolyzed protein mixed with spinosad exhibited higher toxicity to adults of *A. fraterculus* (Diptera: Tephritidae), depending on the food attractant used. Using toxic bait formulated with the food attractant Noctovi^®^ 43SB, Justiniano and Fernandes [30] reported 100% mortality when methomyl was employed as the lethal agent. Likewise, Liu et al. [70] found 100% mortality for *H. armigera*, *Agrotis ipsilon* (Hufnagel), and *Spodoptera litura* (Fabricius) (Lepidoptera: Noctuidae) in toxic baits formulated with indoxacarb, spinosad, and methomyl. A similar pattern was observed by Mensah et al. [71], who evaluated insecticides suitable for formulations containing the attractant Magnet^®^ and identified spinosad, methomyl, and thiodicarb as the most promising options.

The use of toxic bait as a control tool for lepidopteran pests has been practiced in several regions around the world, resulting in reduced damage caused by immature stages and increased crop productivity. In China, Wang et al. [39] applied toxic bait infused with the insecticide methomyl—commercially known as the bio-bait Bioattract^®^-Heli—and observed a reduction in *H. armigera* populations and egg numbers, as well as a 6–8% increase in maize yield. Similarly, Mensah and MacPherson [72] reported a decrease in the population density of *Helicoverpa* species in Bollgard II^®^ cotton fields using thiodicarb as the lethal agent in attract-and-kill formulations with the Magnet^®^ attractant. The authors emphasized that treated areas contributed to reducing the abundance of *Helicoverpa spp*. in adjacent untreated conventional cotton fields (controls). According to Gregg et al. [37], the combination of Magnet^®^ and thiodicarb, when used in curative management programs, reduced *H. armigera* population density by 50%. Moreover, preventive management programs resulted in a 90% reduction. A similar pattern was observed by Gregg et al. [37] in studies conducted in Australia, demonstrating that the use of toxic baits as a management tool for *H. armigera* helped to suppress population peaks originating from previous cropping seasons, and contributed to a reduction in egg numbers and damage caused by immature stages.

The control effectiveness of toxic bait depends on its attractiveness and the toxicity of the insecticidal agent. However, characteristics related to the lethal agent—such as (a) efficacy at low concentrations, (b) absence of repellent or deterrent effects on insect behavior, (c) persistence compatible with the attractiveness of the bait, (d) minimal or no impact on non-target organisms, and (e) rapid incapacitation and death of exposed insects—are essential criteria when selecting an insecticide for toxic bait formulations [44,68]. Additionally, attributes of the attractant, including the matrix of the formulated product, the composition of the attractant blend, and the release density of the semiochemicals (pheromones or kairomones), contribute to the success of this management strategy, as inadequate levels of these components compromise the ability of semiochemicals to attract insects and induce contact with the toxic bait formulations [73].

The toxic potential of the lethal agent used in toxic bait formulations depends on its concentration, as low insecticide concentrations reduce bait effectiveness and allow the survival of adult insects [74]. In the present study, methomyl exhibited the highest toxicity to *S. frugiperda* adults, followed by indoxacarb and spinetoram. In studies conducted by Del Socorro et al. [68], concentrations of methomyl ranging from 0.015 to 0.5% (*v v*^−1^ a.i.) resulted in mortality rates above 80% for *H. armigera* adults. In the same study, the authors also reported 100% mortality for thiodicarb (0.005–0.5%), spinosad (0.04–0.16%), and indoxacarb (0.10% *v v*^−1^ a.i.). According to Revis et al. [75], evaluations of the toxic bait GF-120 at different dilutions showed no significant difference in mortality of *Bactrocera cucurbitae* (Coquillett) (Diptera: Tephritidae) adults at concentrations up to 10 ppm of spinosad (mortality > 80%). In the study by [70], 100% mortality of *H. armigera* adults was recorded for chlorantraniliprole, emamectin benzoate, indoxacarb, thiodicarb, spinetoram, and spinosad at a concentration of 100 mg a.i. L^−1^ of toxic bait. In the same study, the authors also observed 100% mortality of *S. litura* adults exposed to chlorantraniliprole, emamectin benzoate, and spinosad at 1.0 mg a.i. L^−1^. Using the Bioattract^®^ + chlorantraniliprole toxic bait at concentrations of 0.25 and 0.125 µL mL^−1^, Zhang et al. [76] reported adult mortality of *H. armigera* of 75.7% and 67%, respectively. The use of toxic bait formulations with low concentrations of active ingredient may result in reduced control efficiency due to the lower amount of food attractant ingested. Additionally, formulations with low concentrations require more time to incapacitate adults, allowing oviposition to occur. From an operational standpoint, toxic bait formulations with insufficient incapacitating concentrations for adults tend to exhibit shorter residual activity, requiring more frequent reapplications depending on environmental conditions [74,76,77].

In our residual activity bioassays, toxic bait formulated with malathion showed reduced efficacy, with 60% mortality at 30 days after application (DAA). However, formulations containing, emamectin benzoate, indoxacarb, chlorantraniliprole, thiodicarb, lambda-cyhalothrin, and chlofenapyr achieved mortality rates above 90% at the same evaluation period. It is important to note that chlorfenapyr exhibited fluctuations in mortality at the later aging intervals evaluated. This pattern is likely associated with the insecticide’s mode of action, as reported in previous studies assessing the residual effects of chlorfenapyr-based formulations. In addition, the residual performance of chlorfenapyr may be influenced by multiple biotic and abiotic factors, including compound degradation mediated by plant metabolic activity, climatic conditions, the mechanism of action of the product, and the species evaluated [78,79,80,81]. It is important to note that these mortality rates may be lower under field conditions, as the present bioassays were conducted in a greenhouse and thus did not experience environmental factors typical of open-field environments. Supporting our findings, Del Socorro et al. [68] observed a reduction in the residual biological activity (mortality) of a toxic bait formulation containing methomyl used to manage *H. armigera* and *Helicoverpa punctigera* (Wallengren) (Lepidoptera: Noctuidae) in cotton fields, with activity declining after four days following application. A similar pattern was reported by White et al. [82], where a toxic bait formulation (5% sugar + 1% powdered milk) mixed with methomyl or spinosad showed a 70% reduction in residual biological activity after 21 days. Likewise, Varikou et al. [83] documented a decrease in the residual biological activity (due to declining active ingredient concentration) of cypermethrin, lambda-cyhalothrin, thiacloprid + deltamethrin, and spinosad used in toxic bait formulations for the control of *Bactrocera oleae* (Rossi) (Diptera: Tephritidae) after eight weeks. Moreover, Gazit et al. [84] found a marked reduction in the biological activity of GF-120 toxic bait after a 10-day aging period, with mortality falling below 20% for the control of *C. capitata*.

Toxic baits used in attract-and-kill systems undergo alterations in their attractive capacity after periods of exposure to environmental conditions when compared with fresh bait formulations [85]. After long durations of exposure, toxic baits exhibit reduced release rates of volatile compounds present in the formulations, degradation of the insecticidal active ingredient, and accumulation of dust particles on the surface, all of which diminish their efficiency [50]. According to Peregrine [86] and Gazit et al. [84], environmental, ecological, and operational factors may compromise the effectiveness of toxic baits in the field. The authors also emphasize that the size of the bait droplet influences performance: smaller droplets (less than 5.0 mm in diameter) show a greater reduction in attractiveness and biological activity within 10 days (droplets > 5.0 mm, 58% mortality; <5.0 mm, 22% mortality). The decline in toxic bait efficiency may also be associated with ecological factors (non-target insects feeding on the bait) and environmental factors (accelerated degradation of the active ingredient). Another important aspect contributing to reduced bait efficiency is the degradation of attractant and feeding stimulation components over time, which compromises bait efficacy [87]. According to Revis et al. [75], the reduction in bait attractiveness may occur due to bait aging as well as environmental factors such as relative humidity, temperature, and rainfall. Under such conditions, Charmilot et al. [88] recommend that, depending on the regions where attract-and-kill technologies—particularly toxic baits—are deployed, additional applications may be necessary due to high temperatures, intense solar radiation, and dust accumulation, which reduce control efficiency.

Toxic bait formulations as a pest management tool for major agricultural commodities can greatly enhance the sustainability of production systems. Their use supports the conservation of biological control agents because the insecticidal active ingredient is applied only to restricted portions of the crop area, thereby reducing the need for full-field spray applications. Moreover, the food-based attractants used in these formulations contain compounds that are repellent to pollinators and to predatory and parasitoid insects, further contributing to the selectivity of the approach. According to Muthomi et al. [45], the use of toxic baits formulated with more selective insecticides (e.g., spinosad) suppressed *P. xylostella* populations without affecting the abundance of beneficial insects (parasitoids and predators) in the studied areas. The first generation of the attractant Noctovi^®^ 43SB (Noctovi) showed no attractiveness to *Apis mellifera* (Linnaeus) (Hymenoptera: Apidae) [89], demonstrating its potential as a promising pest management tool for Noctuidae species without impairing ecosystem services. Within the attract-and-kill framework, toxic baits effectively remove both sexes of the target pest from the cropping system—particularly mated females—thereby reducing the number of offspring in the subsequent generation and, consequently, crop damage [37,39,41]. However, further studies are needed to strengthen the use of toxic baits as a pest management tool, particularly applied research aimed at providing the scientific community with information on medium- and long-term effects on beneficial insect communities, as well as on resistance evolution or suppression to synthetic insecticides and *Bt* crops.

## 5. Conclusions

Based on our findings in conditions of laboratory, food-attractant-based toxic baits represent a promising strategy for the management of *S. frugiperda* within an IPM framework. Among the insecticides tested, methomyl, indoxacarb, spinetoram, thiodicarb, chlorantraniliprole, and emamectin benzoate showed strong potential for incorporation into toxic bait formulations with the food attractant Noctovi^®^ 43SB. In greenhouse conditions, these mixtures maintained residual activity for up to 30 days after application (DAA) under rain-free conditions, achieving more than 80% mortality of the evaluated insects. Collectively, these results underscore the value of attract-and-kill systems as an efficient, selective, and environmentally compatible tool for improving pest management outcomes while supporting sustainable agricultural production. Indeed, further field-based studies are required to assess the effectiveness of this management tool, as well as to develop application programs for toxic baits that preserve the efficiency of the technique, ensuring consistent suppression and effective population control of the pest.

## Figures and Tables

**Table 1:** Commercial insecticides used at the recommended dose for the larval stage in toxic bait formulations in association with the food attractant Noctovi^®^ 43SB for adult *S. frugiperda* insects.

Active Ingredient (a.i.)	Commercial Trade	Manufacture	Concentration	Dose ^a^		Concentration (g a.i. L^−1^)	Chemical Group ^b^
				a.i.	p.c.		
*Allium sativum* ^i^	Vigga^®^	Omex Agrifluids do Brazil, Piracicaba, SP, Brazil	800	320	400	16.0	Plant extract
Emamectin benzoate ^ii^	Proclaim^®^ 50	Syngenta Proteção de Cultivos Ltda, Paulínia, SP, Brazil	50	10	200	1.0	Avermectin (6)
Chlorantraniliprole ^iii^*	Premio^®^	FMC Química do Brasil Ltda, Campinas, SP, Brazil	200	13.3	66.6	4.0	Anthranilic diamides (28)
Chlorfenapyr ^iv^*	Pirate^®^	Basf S/A, São Paulo, SP, Brazil	240	125	500	4.8	Pyrazole analog (13)
Chlorpyrifos ^v^	Clorpirifós Nortox EC	Nortox S/A, Arapongas, PR, Brazil	480	192	400	9.6	Organophosphate (1B)
Spinetoram ^vi^ *	Delegate^®^	Dow AgroSciences Industria Ltda, São Paulo, SP, Brazil	250	3.0	12	5.0	Spinosyns (5)
Spinosad ^vii^ *	Tracer^®^	Dow AgroSciences Industria Ltda, São Paulo, SP, Brazil	480	24	50	9.6	Spinosyns (5)
Fipronil ^viii^	Fipronil Nortox	Nortox S/A, Arapongas, PR, Brazil	250	50	200	5.0	Pyrazole (2B)
Indoxacarb ^ix^ *	Avatar^®^	FMC Química do Brasil Ltda, Campinas, SP, Brazil	150	24	160	3.0	Oxadiazine (22A)
Lambda-cyhalothrin ^x^	Karate Zeon 50 SC	Syngenta Proteção de Cultivos Ltda, Paulínia, SP, Brazil	50	5	100	1.0	Pyrethroid (3A)
Malathion ^xi^ *	Malathion 1000 EC	FMC Química do Brasil Ltda, Campinas, SP, Brazil	1000	1000	1000	20.0	Organophosphate (1B)
Methomyl ^xii^ *	Lannate^®^ BR	DuPont do Brasil, Barueri, SP, Brazil	215	43	200	4.3	Carbamate (1A)
Novaluron ^xiii^	Rimom Supra 100 SC	ADAMA Brasil S/A, Londrina, PR, Brazil	100	15	150	2.0	Benzoylurea (15)
Profenofos + Cypermethrin ^xiv^	Polytrin^®^	Syngenta Proteção de Cultivos Ltda, Paulínia, SP, Brazil	400 + 40	44	100	8.8	Organophosphate (1B) + Pyrethroid (3A)
Thiodicarb ^xv^ *	Larvin WG	Bayer CropScience Ltda, São Paulo, SP, Brazil	800	40	50	16.0	Carbamate (1A)
Zeta-cypermethrin ^xvi^ *	Mustang^®^ 350 EC	FMC Química do Brasil Ltda, Campinas, SP, Brazil	350	14	40	7.0	Pyrethroid (3A)

^a^ dose of comercial product recorded for control of ^ii, iii, iv, v, ix, x, xii, xiii, xiv, xv, xvi^ *S. frugiperda* (Corn); ^i^ *Meloidogyne incognita* (Soybean); ^vi, vii^ *Spodoptera eridania* (Corn); ^xi^ *Anticarsia gemmatalis* (Soybean); ^viii^ *Elasmopalpus lignosellus* (Corn) (Brazil 2018) in g a.i. and g or mL of the commercial product (p.c.) per 100 L of water. ^b^ Chemical group of insecticides according to the IRAC mode-of-action (MoA) classification * Insecticides used in the “Toxicity and Residual Effect bioassays”.

**Table 2:** Lethal concentrations (LC_50_ and LC_90_; in mg. a.i. L^−1^) of toxic bait formulations on *Spodoptera frugiperda* adults (laboratory bioassay: temperatures of 25 ± 2 °C, relative humidity of 70 ± 10%).

Insecticide	Slope ± SE	LC_50_ (CI 95%) ^a^	LC_90_ (CI 95%) ^a^	χ^2 b^	df ^c^
Methomyl	2.82 ± 0.14	322.0 (215.0–473.0) a	1160.0 (1370.0–1740.0) a	8.04	6
Spinetoram	2.91 ± 0.11	816.0 (675.00–1002.0) b	3648.0 (3570.0–4100.0) c	9.02	6
Spinosad	3.04 ± 0.24	5280.0 (6620.0–7240.0) c	7296.0 (6760.0–8010.0) c	8.18	6
Malathion	3.35 ± 0.26	2000.0 (1400.0–2240.0) a	9000.0 (7500.0–9600.0) a	9.85	6
Thiodicarb	3.56 ± 0.38	7760.0 (6240.0–9280.0) c	16,800.0 (16,240.0–18,800.0) d	9.78	6
Indoxacarb	2.91 ± 0.35	810.0 (600.0–1300.0) b	2610.0 (2550.0–2830.0) b	7.83	6
Chlorantraniliprole	3.04 ± 0.44	920.0 (800;0–1020.0) b	3740.0 (3500.0–4200.0) b	7.07	6
Clofenapyr	2.90 ± 0.43	2320.0 (2010.0–2640.0) c	4530.0 (3740.0–5040.0) b	8.11	6
Zeta-cypermethrin	3.11 ± 0.32	3040.0 (2620.0–3470.0) c	7520.0 (7170.0–7660.0) d	9.05	6

^a^ LC_50_ and LC_90_: Lethal Concentration (mg a.i. L^−1^) required to kill 50 and 90% of *S. frugiperda* adults, values designated by different letters within a column are significantly different from each other through non-overlap of 95% fiducial limits. Significance of differences among slopes determined by likelihood ratio test of equality followed by pairwise comparisons using non-overlapping fiducial limits; CI 95%: 95% Confidence Interval; ^b^ χ^2^: Pearson’s Chi-squared value; ^c^ df: Degrees of freedom.

**Table 3:** Average mortality (%) of *Spodoptera frugiperda* adults 120 h after exposure to toxic baits on cotton leaves in different intervals after spraying (conditions greenhouse: temperatures of 25 ± 0.19 °C, relative humidity of 58.16 ± 0.62%).

Treatments (g a.i/L^−1^ Food Attractant)	3 DAA		7 DAA		15 DAA		21 DAA		30 DAA		*p* Values
	N ± SE ^1^	M% ^2^	N ± SE ^1^	M%	N ± SE ^1^	M%	N ± SE ^1^	M%	N ± SE ^1^	M%	
Methomyl (4.3)	0.0 ± 0.00 Ba	100.00	0.0 ± 0.0 Ba	100.0	0.0 ± 0.0 Ca	100.0	0.6 ± 0.1 Ca	95.0	2.2 ± 0.2 Bb	81.7	F = 4.11; df = 4; 16; *p* < 0.001
Spinosad (9.6)	0.0 ± 0.0 Ba	100.00	0.0 ± 0.0 Ba	100.0	0.6 ± 0.1 Ca	95.0	0.2 ± 0.1 Ca	98.0	3.6 ± 0.5 Ab	70.0	F = 6.77; df = 4; 16; *p* < 0.001
Zeta-cypermethrin (7.0)	2.4 ± 0.3 Aa	80.0	2.6 ± 0.4 Aa	78.3	2.0 ± 0.2 Ba	83.3	1.8 ± 0.4 Ba	85.0	3.2 ± 0.6 Aa	73.3	F = 6.90; df = 4; 16; *p* < 0.001
Clofenapyr (4.8)	0.6 ± 0.1 Ba	95.0	0.6 ± 0.2 Ba	95.0	0.6 ± 0.1 Ca	95.0	1.4 ± 0.3 Bb	88.3	0.4 ± 0.1 Ca	96.7	F = 5.16; df = 4; 16; *p* < 0.001
Emamectin benzoate (1.0)	0.4 ± 0.1 Ba	96.7	0.4 ± 0.1 Ba	96.0	0.0 ± 0.0 Ca	100.0	0.2 ± 0.1 Ca	98.3	0.0 ± 0.0 Ca	100.0	F = 8.76; df = 4; 16; *p* < 0.001
Spinetoram (5.0)	0.2 ± 0.1 Ba	98.3	0.2 ± 0.0 Ba	98.0	0.4 ± 0.1 Ca	96.0	0.4 ± 0.1 Ca	96.0	2.0 ± 0.3 Bb	83.3	F = 7.89; df = 4; 16; *p* < 0.001
Indoxacarb (3.0)	0.6 ± 0.1 Ba	94.0	0.4 ± 0.1 Ba	96.0	0.2 ± 0.1 Ca	98.0	0.2 ± 0.1 Ca	98.0	0.2 ± 0.1 Ca	98.0	F = 10.33; df = 4; 16; *p* < 0.001
Chlorantraniliprole (4.0)	0.4 ± 0.1 Ba	96.0	0.4 ± 0.1 Ba	96.0	0.6 ± 0.2 Ca	94.0	0.8 ± 0.2 Ca	92.0	0.8 ± 0.2 Ca	92.0	F = 9.21; df = 4; 16; *p* < 0.001
Thiodicarb (16.0)	0.0 ± 0.0 Ba	100.0	0.4 ± 0.2 Ba	96.0	0.2 ± 0.1 Ca	98.0	0.2 ± 0.1 Ca	98.0	0.2 ± 0.1 Ca	98.0	F = 7.13; df = 4; 16; *p* < 0.001
Lambda-cyhalothrin (16.0)	0.6 ± 0.2 Ba	94.0	0.6 ± 0.1 Ba	94.0	1.4 ± 0.5 Cb	86.0	1.4 ± 0.5 Bb	86.0	0.8 ± 0.2 Ca	92.0	F = 8.25; df = 4; 16; *p* < 0.001
Malathion (20.0)	0.2 ± 0.1 Ba	98.0	2.8 ± 0.6 Ab	78.0	6.6 ± 1.1 Ac	44.0	6.4 ± 1.0 Ac	46.0	4.6 ± 0.8 Ac	54.0	F = 9.10; df = 4; 16; *p* < 0.001
*F*	12.45		17.11		10.45		18.10		9.04		
*df*	10; 55		10; 55		10; 55		10; 55		10; 55		
*p*	0.001		0.001		0.001		0.001		0.001		

^1^ Means followed by uppercase letters in the column and lowercase letters in the row do not differ by the Tukey test at 0.05% significance level. ^2^ Mortality corrected by the Henderson and Tilton [53] formula. N: Average number of live adults.

## Data Availability

The original contributions presented in the study are included in the article; further inquiries can be directed to the corresponding author.

## References

[1] Montezano D.G., Specht A., Saosa-Gómez D.R., Roque-Specht V.F., Sousa-Silva J.C., Paula-Moraes S.V., Peterson J.A., Hunt T.E. (2016). Host plants of *Spodoptera frugiperda* (Lepidoptera: Noctuidae) in the Americas. Afr. Entomol..

[2] Barros E.M., Torres J.B., Ruberson J.R., Oliveira M.D. (2010). Development of *Spodoptera frugiperda* on different hosts and damage to reproductive structes in cotton. Entomol. Exp. Appl..

[3] Nagoshi R.N., Meagher R.L., Flanders K., Gore J., Jackson R., Lopez J., Armstrong J.S., Buntin D., Sansone C., Leonard B.R. (2008). Using halotypes to monitor the migratory of fall armyworm (Lepidoptera: Noctuidae) corn–strain population from Texas and Florida. J. Econ. Entomol..

[4] Bueno R.C.O.F., Bueno A.F., Moscardi F., Parra J.R.P., Hoffman-Campo C.B. (2011). Lepidopteran larva consumption of soybean foliage: Basis for development multiple-species economic theresholds for pest management decisions. Pest. Manag. Sci..

[5] Farias J.R., Andow D.A., Horikoshi R.J., Sorgatto R.J., Fresia P., Santos A.C., Omoto C. (2014). Field-evolved resistance to Cry1F maize by *Spodoptera frugiperda* (Lepidoptera: Noctuidae) in Brazil. Crop Prot..

[6] Nagoshi R.N., Fleischer S., Meagher R.L. (2009). Texas is the overwintering source of fall armyworm in central Pennsylvania: Implications for migrations into the northeastern United States. Environ. Entomol..

[7] Nagoshi R.N., Rosas-García N.M., Meagher R.L., Fleischer S.J., Westbrook J.K., Sappington T.W., Murúa G.M. (2015). Haplotype profile comparisons between *Spodoptera frugiperda* (Lepidoptera: Noctuidae) populations from Mexico with those from Puerto Rico, South America, and the United States and their implications to migratory behavior. J. Econ.Entomol..

[8] Goergen G.P., Kumar P.L., Snkung S.B., Togola A., Tamó M. (2016). First report of outbreaks of the fall armyworm *Spodoptera frugiperda* (J. E. Smith) (Lepidoptera: Nocutidae); a new alien invasive pest in West and Central Africa. PLoS ONE.

[9] Njuguna E., Nethononda P., Maredia K., Mbabazi R., Kachapuluda P., Rowe A., Ndolo D. (2021). Experiences and perspectives on *Spodoptera frugiperda* (Lepidoptera: Noctuidae) management in Sub-Saharan Africa. J. Integr. Pest. Manage.

[10] Baloch M.N., Fan J., Haseeb M., Zhang R. (2020). Mapping potential distribution of *Spodoptera frugiperda* (Lepidoptera: Noctuidae) in central Asia. Insects.

[11] Song Y., Zhang H., Wu K. (2024). Transboudary migration of *Spodoptera frugiperda* between China and the South-Southeast Asian region. J. Pest Sci..

[12] Tay W.T., Rane R., Padovan A., Walsh T., Elfekih S., Downes S., Nam K., Alencon E.D., Zhang J., Wu Y. (2022). Global population genomic signature of *Spodoptera frugiperda* (fall armyworm) supports complex introduction events across the Old World. Commun. Biol..

[13] Szanyi S., Barta M., Velchev D., Beshkov S., Mumford S., Todorov I., Nagy A., Varga Z., Tóth M., Toshova T. (2025). First report of a migratory pest, the fall armyworm, *Spodoptera frugiperda* (JE Smith, 1797) (Lepidoptera, Noctuidae) from Bulgaria. Insects.

[14] Kumar R.M., Gadratagi B.G., Paramesh V., Kumar P., Madivalar Y., Narayanappa N., Ullah F. (2022). Sustainable management of invasive fall armyworm, *Spodoptera frugiperda*. Agronomy.

[15] Carvalho R.A., Omoto C., Field L.M., Williamson M.S., Bass C. (2013). Investing the molecular mechanisms of organophasphate and pyretoid resistance in the fall armyworm *Spodoptera frugiperda*. PLoS ONE.

[16] Burtet L.M., Bernardi O., Melo A.A., Pes S.M., Strahl T.T., Guedes J.V.C. (2017). Managing fall armyworm, *Spodoptera frugiperda* (Lepidoptera: Noctuidae), with Bt maize and insecticides in southerm Brazil. Pest. Manag. Sci..

[17] Horikoshi R.J., Bernardi D., Bernardi O., Malaquias J.B., Okuma D.M., Miraldo L.L., Amaral F.S.A., Omoto C. (2016). Effective dominace of resistance of *Spodoptera frugiperda* to Bt maize and cotton varieties: Implications for resistance management. Sci. Rep..

[18] Bolzan A., Padovez F.E.O., Nascimento A.R.B., Kaiser I.S., Lira E.C., Amaral F.S.A., Kanno R.H., Malaquias J.B., Omoto C. (2019). Selection and characterization of the inheritance of resistance of *Spodoptera frugiperda* (Lepidoptera: Noctuidae) to chlorantraniliprole and cross-resistance to other diamide insecticides. Pest. Manag. Sci..

[19] Paredes-Sánchez F.A., Rivera G., Bocanegra-García V., Martínez-Padrón H.Y., Berrones-Morales M., Niño-García N., Herrera-Mayorga V. (2021). Advances in control strategies against *Spodoptera frugiperda*. A review. Molecules.

[20] Van den Berg J., Du Plessis H. (2022). Chemical control and insecticide resistance in *Spodoptera frugiperda* (Lepidoptera: Noctuidae). J. Econ. Entom..

[21] Ngegba P.M., Khalid M.Z., Jiang W., Zhong G. (2025). An overview of insecticide resistance mechanisms, challenges, and management strategies in *Spodoptera frugiperda*. Crop Prot..

[22] Boaventura D., Ulrich J., Lueke B., Bolzan A., Okuma D., Gutbrod O., Zeng Q., Dourado P.M., Martinelli S., Flagel L. (2020). Molecular charactezation of Cry1F resistance in fall armyworm; *Spodoptera frugiperda* from Brazil. Insect. Biochem. Mol. Biol..

[23] Omoto C., Bernardi O., Salmeron E., Sorgatto J.R., Dourado P.M., Crivellari A., Carvalho R.A., Willse A., Martinelli S., Head G.P. (2016). Field-evolved resistance to Cry1Ab maise by *Spodoptera frugiperda* in Brazil. Pest. Manag. Sci..

[24] Bernardi D., Salmeron E., Horikoshi R.J., Bernardi O., Dourado P.M., Carvalho R.A., Martinelli S., Head G.P., Omoto C. (2015). Cross-resistance between Cry1 proteins in fall armyworm (*Spodoptera frugiperda*) may affect the durability of current pyramided Bt maize hybrids in Brazil. PLoS ONE.

[25] Yang F., Williams J., Huang F., Kerns D.L. (2022). Genetc basis and cross-resistance of Vip3Aa resistance in *Spodoptera frugiperda* (Lepidoptera: Noctuidae) derived from Texas, USA. Crop Prot..

[26] Silva A.F.T., Silva L.B., Malaquias J.B., Salustiano A.S., Correia Neto D.F., Pacheco D., Fragosos D.B., Pereira E.J.G. (2024). Susceptibility of fall armyworm field populations to Vip3Aa/Cry Bt maize in a tropical agricultural region. Agronomy.

[27] Harrison R.D., Thiefelder C., Baudron F., Chiwada P., Midega C., Schaffner U., Van Den Berg J. (2019). Agro-ecological options for fall armyworm (*Spodoptera frugiperda* JE Smith) management: Providing low-cost; smallholder friendly solutions to an invasive pest. J. Environ. Manag..

[28] Howse P.E., Stevens I.D.R., Jones O.T. (1998). Insect’s Pheromones and Their Use in the Pest Management.

[29] Zarbin P.H.G., Rodrigues M.A.C.M., Lima E.R. (2009). Feromônios de insetos: Tecnologia e desafios para uma agricultura competitiva no Brasil. Quim. Nova.

[30] Justiniano W., Fernandes M.G. (2020). Effect of food attractants and insecticides toxicity for the control of *Spodoptera frugiperda* (Lepidoptera: Noctuidae) adults. J. Agric. Sci..

[31] Camelo L.A., Landolt P.J., Zack R.S. (2007). Kairomones based attract-and-kill system effective against alfafa looper (Lepidoptera: Noctuidae). J. Econ. Entomol..

[32] Assefa F., Ayalew D. (2019). Status and control measures of fall armyworm (*Spodoptera frugiperda*) infestations in maize fields in Ethiopia: A review. Congent. Food. Agric..

[33] Landolt P.J., Ohler B., Lo P., Cha D., Davis T.S., Suckling D.M., Brunner J. (2014). N-Butyl sulfide as an attractant and coattractant for male and female codling moth (Lepidoptera: Tortricidae). Environ. Entomol..

[34] Gregg P.C., Del Socorro A.P., Hawes A.P., Binns M.R. (2016). Development bisexual attract-and-kill for polyphagous insects: Ecological rationale versus pragmatics. J. Chem. Ecol..

[35] Gregg P.C., Del Socorro A.P., Landolt P.J. (2018). Advances in attract-and –kill for agricultural pests: Beyond pheromones. Annu. Rev. Entomol..

[36] Cardé R.T., Minks A.K. (1995). Control of moth pests by mating disruption successes and constrains. Ann. Rev. Entomol..

[37] Gregg P.C., Del Socorro A.P., Wilson S., Knight K.M., Binns M., Aemytage P. (2022). Bisexual attract-and-kill: A novel component of resistance management for transgenic cotton in Australia. J. Econ. Entomol..

[38] Lunagariya M.V., Zala M.B., Varma H.S., Suthar M.D., Patel M.B., Patel B.N., Borad P.K. (2020). Efficacy of poison baits against fall armyworm, *Spodoptera frugiperda* (J.E. Smith) infesting maize. J. Entomol. Zool. Stud..

[39] Wang L., He L., Zhu X., Zhang J., Li N., Fan J., Sun X., Zhang L., Lin Y., Wu K. (2023). Large-area field application confirms the effectiveness of toxicant-infused bait for managing *Helicoverpa armigera* (Hübner) in maize fields. Pest. Manag. Sci..

[40] Justiniano W., Fernande M.G., Raizer J. (2021). Toxic bait as an alternative tool in the management of *Spodoptera frugiperda* in second corn crops. J. Agric. Sci..

[41] Faleiro J.R., Al-Shawaf A.M., Al-Dandan A.M., Al-Odhayb A., Abdallah A.B., Peixoto M.P., Vargas R., Botton M., Chidi S., Borges R. (2016). Controlled release products from managing insect pests. Outlooks Pest. Manag..

[42] (2017). Isca Tecnologia. https://www.isca.com/downloads/632d24004af25noctovi_rotulo.pdf.

[43] Galm U., Sparks T.C. (2015). Natural product derived insecticides: Discovery and development of spinetoram. J. Ind. Microb. Biotech..

[44] Yee W.L., Alston D.G. (2016). Sucrose mixed with spinosad enhances kill and reduces oviposition of *Rhagoletis indifferens* (Diptera: Tephritidae) under low food availability. J. Entomol. Sci..

[45] Muthomi P.K., Seal D., Mafra-Neto A., Liburd O.E. (2025). A semiochemical attract-and-kill formulations to manage diamondback moth (Lepidoptera: Plutellidae). J. Econ. Entomol..

[46] Haynes K.F. (1988). Sublethal effects of neurotoxic insecticides on insect behaviour. Ann. Rev. Entomol..

[47] Casida J.E., Durkin K.A. (2013). Neuroactive insecticides: Targets, selectivity, resistance, and secondary effects. Ann. Rev. Entomol..

[48] Baronio C.A., Schutze I.X., Nunes M.Z., Bernardi D., Machota Junior R., Bortoli L.C., Arioli C.J., Garcia F.R.M., Botton M. (2019). Toxicities and residual effect of spinosad and alpha-cypermethrin-based baits to replace malathion for *Ceratitis capitata* (Diptera: Tephritidae) control. J. Econ. Entomol..

[49] Piñero J.C., Souder S.K., Vargas R.I. (2010). Comparison of rain-fast bait stations versus foliar baits spray for control fruit fly *Bractocera dorsalis* in papaya orchards in Havaii. J. Insect. Sci..

[50] Losel P., Potting R., Ebbinghaus D., Scherkenbeck J. (2002). Factors affecting the field performance of attracticidae against the codling moth *Cydia pomonella*. Pest Manag. Sci..

[51] Greene G.L., Lepla N.C., Dickerson W.A. (1976). Velvetbean caterpillar: A rearing procedure and artificial medium. J. Econ. Entomol..

[52] SAS Institute (2011). Statistical Analysis System: Getting Started with the SAS Learning.

[53] Henderson C.F., Tilton E.W. (1955). Tests with acaricides against the brown wheat mite. J. Econ. Entomol..

[54] Baldin M.M., Schutze I.X., Baronio C.A., Garcia F.R.M., Botton M. (2018). Concentration and lethal time of toxic baits based on spinosyns on *Ceratitis capitata* and *Diachasmimorpha longicaudata*. Pesq. Agropec Trop..

[55] Muthomi P.K., Rhodes E., Liburd O.E. (2025). Evalution of a SPLAT^®^-based semiochemical with insecticides form diamondback moth management in cabbage. Arthop. Manag. Tests.

[56] Villavaso E.J., Mulrooney J.E., McGovern W.L. (2003). Boll weevil (Coleoptera: Curculionidae) bait sticks: Toxicity and malathion content. J. Econ. Entomol..

[57] Kruger A.P., Ramos R.S., Favoreto A.L., Tran K., Broms K., Andrade T., Galvan T. (2025). Redução da população de *Spodoptera frugiperda* (J.E. Smith, 1779) (Lepidoptera: Noctuidae) com manejo comportamental na cultura da soja. Bioassay.

[58] Murtiningsih R., Moekasan T.K., Prabaningrum L., Gunadi N., Setiawati W., Muharam A., Hsyim A., Udiarto B.K., Sulastini I., Gunaemi N. (2024). Light traps based-control threshold: An alternative method for hot pepper pests’management. Chil. J. Agric. Res..

[59] Sarkar S.C., Wang E., Wu S., Lei Z. (2018). Application of trap cropping as companion plants for the management of agricultural pest: A review. Insects.

[60] Stupp P., Machota Junior R., Cardoso T.D.N., Padilha A.C., Hoffer A., Bernardi D., Botton M. (2021). Mass trapping is a viable alternative to insecticides for management of *Anastrepha fraterculus* (Diptera: Tephritidae) in apple orchard in Brazil. Crop Prot..

[61] Bird L.J., Drynan L.J. (2023). Comparison of insecticide toxicity in adult and larval stages of *Spodoptera frugiperda* (J.E. Smith) and *Helicoverpa armigera* (Hunber) (Lepidoptera: Noctuidae). Crop Prot..

[62] Nestel D., Nemny-Lavy E., Zilberg L., Weiss M., Akiva R., Gazit Y. (2004). The fruit fly PUB: A phagostimulation unit bioassey system to quantitatively measure ingestion of bait by individual flies. J. Appl. Entomol..

[63] Gazit Y., Akiva R. (2017). Toxicity of malathion and Spinosad to *Bactrocera zonata* and *Ceratitis capitata* (Diptera: Tephritidae). Fla. Entomol..

[64] Rharrabe K., Jacquin-Joly E., Marion-Poll F. (2014). Electrophysiological and behavioral response of *Spodoptera littoralis* caterpillars to attractive and repellent plant volatiles. Front. Ecol. Evol..

[65] Rodruiguez-Saona C., Wanumen A.C., Salamanca J., Holdcraft R., Kyryczenko-Roth V. (2016). Toxicitiy of insecticides on various life stages of two tortricid pests of Cranberries and on a non-target predator. Insects.

[66] Leonova I.N., Slynko N.M. (1996). Comparative study of insecticide susceptibility and activities of detoxifying enzymes in larvae and adults of cotton bollworm. Ins. Bioch. Phys..

[67] Forrester N.W., Cachill M., Bird L.J., Layland J.K. (1993). Management of pyretroid and endosulfan resistance in *Helicoverpa armigera* (Lepidoptera: Noctuidae) in Autralia. Bull. Entomol. Res. Suppl..

[68] Del Socorro A.P., Gregg P.C., Hawes A.J. (2010). Development of a synthetic plant volatile-based attracticide for female noctuid moth. III. Insecticides for adult *Helicoverpa armigera* (Hübner) (Lepidoptera: Noctuidae). Aust. J. Entomol..

[69] Schutze I.X., Baronio C.A., Baldin M.M., Loek A.E., Botton M. (2018). Toxicity and residual effects of toxic baits with spinosyns on the South American fruit fly. Pesq. Agropec. Bras..

[70] Liu Y., Gao Y., Liang G., Lu Y. (2017). Chlorantraniliprole as a candidate pesticide used in combination with the attracticides for lepidopteran moths. PLoS ONE.

[71] Mensah R.K., Gregg P.C., Del Socorro A.P., Moore C.J., Hawes A.J., Watts N. (2013). Integrated pest management in cotton: Exploiting behaviour-modifying (semiochemical) compounds for managing cotton pests. Crop Pasture. Sci..

[72] Mensah R.K., Macpherson I. (2010). Lure-and-kill as reduced-risk strategy for managing *Helicoverpa* spp. on conventional cotton crops with transgenic cotton fields. J. Biol. Control..

[73] El Sayed A.M., Suckling D.M., Byers J.A., Jang E.B., Wearing C.H. (2009). Potential of “lure and kill” in long–term pest management and erradication of invasive species. J. Econ. Entomol..

[74] Mangan R., Moreno D.S., Thompson G.D. (2006). Bait dilution spinosad concentration and efficacy of GF-120 based fruit fly sprays. Crop Prot..

[75] Revis H.C., Miller N.W., Vargas R.I. (2004). Effects of aging and diluition on attraction and toxicity of GF-120 fruit fly bait spray for melon fly control in Hawaii. J. Econ. Entomol..

[76] Zhang Q., Liang H., Lu Y. (2020). Control efficacy of food attractant combined with low dose chlorantraniliprole on *Helicoverpa armigera* population. Chin. J. Biol. Cont..

[77] Harter W.R., Botton M., Nava D.N., Grutzmacher A.D., Gonçalves R.S., Machotta Junior R., Bernardi D., Zanardi O.Z. (2015). Toxicities and residual effects baits containing spinosad or malathion to control the adult *Anastrepha fraterculus* (Diptera: Tephritidae). Fla. Entomol..

[78] Ngufor C., Fongnikin A., Hobbs N., Gbegbo M., Kiki L., Odjo A., Akogbeto M., Rowland M. (2020). Indoor spraying with chlofenapyr (a pyrrole insecticide) provides residual control of pyrethroid-resistant malaria vectors ins southern Benin. Malar. J..

[79] Che-Mendoza A., González-Olvera G., Medina-Barreiro A., Arisqueta-Chablé C., Bibiano-Marin W., Correa-Morales F., Kirstein O.D., Manrique-Saide P., Vazquez-Prokopec G.M. (2021). Efficacy to targeted indoor residual sprying with to pyrrole insecticides chlofenapyr against pyretroid-resistant *Aedes aegypti*. PLoS Negl. Trop. Dis..

[80] Leite M.I.S., Carvalho G.A., Maia J.B., Makiyama L., Vilela M. (2010). Residual action of insecticides to larvae and adults of the predator *Cycloneda sanguinea* Linnaeus, 1763 (Coleoptera: Coccinelidae). Arq. Inst Biol. São Paulo.

[81] Leonard P.K. (2000). Chlofenapyr, a novel IPM compatible resistance management tool for fruit production. Acta Hort..

[82] White W.H., McCoy C.M., Meyer J.A., Winkle J.R., Plummer P.R., Kemper C.J., Starkey R., Snyder D.E. (2007). Knockdown and mortality comparisons among Spinosad, Imidacloprid, and Methomyl–containing baits against sucetible *Musca domestica* (Diptera: Muscidae) under laboratory conditions. J. Econ. Entomol..

[83] Varikou K., Garanthonakis N., Marketali M., Charalampous A., Anagnostopoulos C., Bempelou E. (2018). Residual degradation and toxicity of insecticides against *Bractocera oleae*. Environ. Sci. Pollut. Res..

[84] Gazit Y., Gavriel S., Akiva R., Timar D. (2013). Toxicity of baited spinosad formulations to *Ceratitis capitata*: From the laboratory to the application. Entomol. Exp. Appl..

[85] Mansour M. (2010). Attract and kill for codling moth *Cydia pomonella* (Linnaeus) (Lepidoptera: Tortricidae) control in Syria. J. Appl. Entomol..

[86] Peregrine D.J. (1973). Toxic baits for the control of pest animals. PANS Pest Artic. News Summ..

[87] Vargas R.I., Miller N.W., Prokopy R.J. (2003). Attraction and feeding responses of mediterranean fruit fly and a natural enemies on protein baits laced with two novel toxins phloxine B and spinosad. Entomol. Exp. Appl..

[88] Charmilot P.J., Hofer D., Pasquier D. (2000). Attract and kill: A new method for control of the codling moth *Cydia pomonella* (L.). Entomol. Exp. Appl..

[89] Borges R., Oliveira da Silva R., Bernardi C., Urrutia W., Mafra-Neto A. (2015). Noctovi, na effective food based attractant for lepidopteran pest. Entomol. Soc. Am..

